# Core–Shell GaAs-Fe Nanowire Arrays: Fabrication Using Electrochemical Etching and Deposition and Study of Their Magnetic Properties

**DOI:** 10.3390/nano12091506

**Published:** 2022-04-28

**Authors:** Eduard V. Monaico, Vadim Morari, Veaceslav V. Ursaki, Kornelius Nielsch, Ion M. Tiginyanu

**Affiliations:** 1 National Center for Materials Study and Testing, Technical University of Moldova, 2004 Chisinau, Moldova; vvursaki@gmail.com (V.V.U.); tiginyanu@asm.md (I.M.T.); 2 Institute of Electronic Engineering and Nanotechnologies “D. Ghitu”, 2028 Chisinau, Moldova; vadimmorari2018@gmail.com; 3 Academy of Sciences of Moldova, 2001 Chisinau, Moldova; 4 Institute for Metallic Materials (IMW), Leibniz Institute of Solid State and Materials Research (IFW Dresden), Helmholtzstr. 20, 01069 Dresden, Germany; k.nielsch@ifw-dresden.de

**Keywords:** nanowires, nanotubes, anodization, electroplating, magnetic anisotropy, hysteresis loop

## Abstract

The preparation of GaAs nanowire templates with the cost-effective electrochemical etching of (001) and (111)B GaAs substrates in a 1 M HNO_3_ electrolyte is reported. The electrochemical etching resulted in the obtaining of GaAs nanowires with both perpendicular and parallel orientations with respect to the wafer surface. Core–shell GaAs-Fe nanowire arrays have been prepared by galvanostatic Fe deposition into these templates. The fabricated arrays have been investigated by means of scanning electron microscopy (SEM) and vibrating sample magnetometry (VSM). The magnetic properties of the polycrystalline Fe nanotubes constituting the shells of the cylindrical structures, such as the saturation and remanence moment, squareness ratio, and coercivity, were analyzed in relation to previously reported data on ferromagnetic nanowires and nanotubes.

## 1. Introduction

Among other nano-platforms, magnetic nanoparticles have been intensively studied during the last years due to their unique magnetic properties, along with other characteristics important for various applications, such as their tunable size, chemical stability, and biocompatibility [1]. When biocompatibility is ensured, the area of biomedical applications is enlarged to magnetic resonance imaging, magnetic hyperthermia treatment, tissue engineering, theranostic, drug delivery systems, cell separation and selection, lab-on-a-chip, etc. [2,3,4,5].

Apart from applications of individual magnetic nanoparticles, arrays of ferromagnetic nanostructures such as wires, tubes, stripes, or rings are of interest in the field of high-density data storage, microelectronics, spintronics, and microwave devices [6,7]. A pronounced shape anisotropy is inherent to these 1-D structures, and their properties are strongly affected by the selected material. Another possibility of tuning their magnetic properties has been widely explored by incorporating segments of different materials or diameters along the length [8,9,10,11,12,13]. Core–shell and tubular arrays offer advantages compared to nanowires, due to the possibilities of varying the thickness of the tube wall in addition to the control of the length and diameter [14,15,16,17].

Nanoscale magnetic structures can be produced with high precision by direct writing assisted by a focused electron beam (FEB), or other lithographic techniques, such as ion beam lithography (IBL) and two-photon lithography [17,18,19]. However, the direct writing method is expensive and its throughput is low. The template-assisted electrodeposition method is much more simple, efficient, low-cost, and widely used for the fabrication of different architectures based on nanowire and nanotube arrays, with excellent control over geometrical features and morphology. Nano-templates with well-defined pore architecture, including those with an ordered arrangement, are used for these purposes. In particular, magnetic arrays with the desired aspect ratio, composition, structure, morphologies, and density are produced to achieve tunable magnetic, magneto-transport, and thermoelectric properties [16].

The most commonly used templates for the fabrication of magnetic arrays are ion track-etched polymers and porous anodic membranes [7,8,16]. Particularly widespread applications were achieved with electrochemical deposition into anodic aluminum oxide (AAO) templates due to their versatile fabrication techniques allowing for the easy tuning of the pore diameter, length, and inter pore distances, as well as modulation of the nanopores diameter with some low-cost modification of the template fabrication process [7]. In spite of these large possibilities to control the parameters of nanomagnetic arrays (nanowires, nanocylinders, and core–shell structures) prepared by template-assisted deposition, they are always oriented perpendicularly to the substrate surface, i.e., they are out of plane. In such a case, they are anisotropic with respect to the plane perpendicular to the template surface, but they are isotropic for the in-plane magnetization. Taking this into account, it was important to develop methods for the preparation of templates with pores oriented parallel to the top surface of the substrate. A technological route was previously elaborated on for this purpose, particularly for semiconductor InP and ZnSe templates [20,21], along with a route for filling these templates with metallic nanotubes or nanodots [22,23].

The goal of this paper is to prepare magnetic nanotube arrays oriented both in-plane and out-of-plane with the substrate on the basis of GaAs nanowire arrays, and to investigate their magnetic properties. The choice of GaAs nanowire arrays is conditioned by the possibilities of producing nanowires oriented both perpendicularly to the substrate, as demonstrated previously on GaAs(111)B substrates [24], and parallel to the substrate, as shown in the present paper on GaAs(001) substrates.

## 2. Materials and Methods

(001)-oriented n-type GaAs wafers and (111)-oriented n-type GaAs wafers with free electron concentrations of 1 × 10^18^ cm^−3^ and 2 × 10^18^ cm^−3^, respectively, cleaved into 1 × 1 cm^−2^ parts, were used in experiments. Before the anodization, the samples were sonicated in acetone for 10 min and rinsed in distilled water and dried, followed by wet chemical etching in HCl/H_2_O with a ratio of (1:3) for 2 min with the aim to remove the native oxide from the surface. The anodization was carried out on the (001) and (111)B surfaces of GaAs in 1 M HNO_3_ electrolyte at applied anodic potential of 4 V. For anodization, the configuration of cell with three electrodes was used. A piece of GaAs sample with a surface of 1 cm^2^ served as working electrode, while a mesh from Pt wire with the total surface 6 cm^2^ was used as counter electrode. The third one was a saturated Ag/AgCl electrode. Etching was performed for 15 min, resulting in a 45 µm thick nanostructured layer containing 45 µm long nanowires with a surface density of approximately 10^8^ nanowires per square centimeter for (111)B GaAs sample, whereas, for (001) GaAs, the length of nanowires could reach 180 µm.

Deposition of Fe inside the prepared nanostructured templates was carried out in the galvanostatic mode at 2 mA/cm^2^ with three-electrode configurations [25]. Bulk or etched GaAs samples served as working electrode (WE). Saturated Ag/AgCl and Pt wires were used as reference (RE) and counter (CE) electrodes, respectively. The time of deposition varied between 10 and 20 s. The process was controlled by computer via a Biologic VSP-128 device (Biologic, Seyssinet-Pariset, France). The samples were kept in NH_3_ for 10 s, and in H_2_O for 10 s before deposition. The electrolyte was freshly prepared and consisted of 0.01 mol/L iron sulfate (FeSO_4_), 0.03 mol/L ammonium sulfate ((NH_4_)_2_SO_4_), and 0.3 mol/L sodium sulfate (Na_2_SO_4_), with pH of 5.1.

The morphology of the prepared samples with and without Fe depositions was studied using a LEO-ZEISS Gemini 1530 (ZEISS, Jena, Germany) scanning electron microscope (SEM), equipped with EDX detector-analyzer.

The magnetization curves of the deposited Fe metallic formations were investigated by a vibrating sample magnetometer (VSM) from Quantum Design VersaLab™ (San Diego, CA, USA) with applied magnetic fields of up to ±3 T at room temperature in both in-plane (ip) and out-of-plane (oop) configurations. The samples were cut in parts with top surfaces of 5 × 5 mm^2^ for VSM measurements.

## 3. Results and Discussion

**Figure 1:**
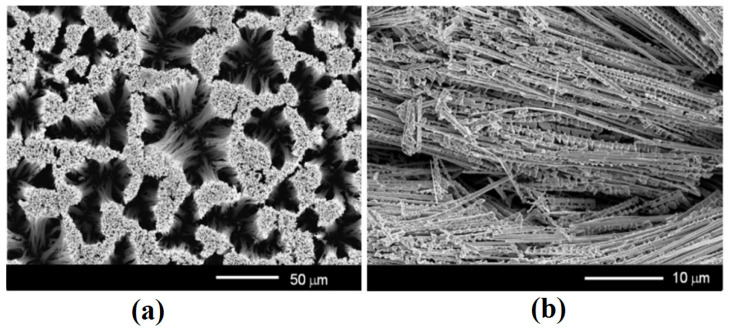
Top view SEM images of anodized GaAs crystals with crystallographic orientations (111)B (**a**) and (001) (**b**) at applied anodization potential of 4 V in 1 M HNO_3_ for 15 min.

The anodization of (111)B substrates in a 1 M HNO_3_ electrolyte for 15 min results in the formation of an array of nanowires (NWs) oriented perpendicular to the substrate surface, similar to those reported recently [24], as shown in Figure 1a, whereas anodization of (001) substrates under the same technological conditions leads to the formation of a nanowire array oriented predominantly parallel to the wafer surface (Figure 1b). As shown in the published paper, the wires produced on the (111)B GaAs wafer are of high crystalline quality, as indicated by narrow XRD reflexes; they preserve the initial (111)B crystallographic orientation of the sample, as indicated by the predominance of (111) and (333) reflexes in the XRD pattern [24].

**Figure 2:**
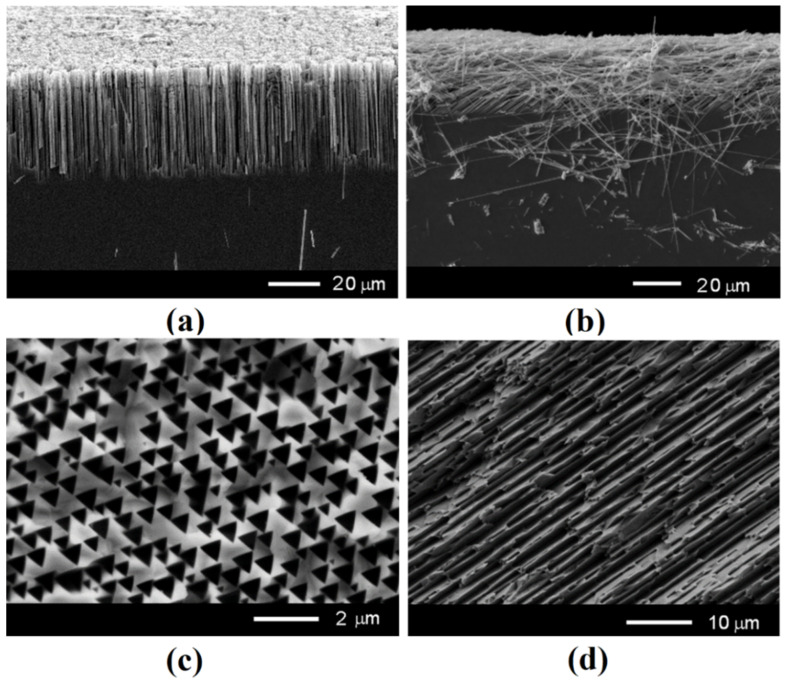
Cross-sectional view of SEM images taken from GaAs nanowires produced on (111)B (**a**) and (001) (**b**) substrates compared with top view SEM images of pores grown in anodized GaAs substrates with (111)B (**c**) and (001) (**d**) orientation.

The mechanism of the formation of nanowires with an orientation predominantly parallel to the substrate surface is supposed to be related to the mechanism of pore growth parallel to the substrate, previously demonstrated in InP and ZnSe compounds [20,21]. Figure 2 compares the formation of nanowires with the growth of pores in GaAs substrates with (111)B and (001) crystallographic orientations. One can see that both the wires and the pores produced on (111)B substrates are perpendicular to the substrate surface, whereas those produced on (001) substrates are predominantly parallel to the substrate surface. The images in Figure 2c,d represent the top view of porous GaAs after successive anodization at an applied potential from 4 V to 3 V of (111)B and (001) GaAs substrates, respectively. The process was followed by sonication for 1 min to disclose the growth direction of pores. From Figure 2c, it can be seen that pores pose a triangular cross-sectional shape due to the formation of crystallographic-oriented pores, in contrast to a round cross-sectional shape inherent to current-line-oriented pores. Note that the formation of pores with the round shape in GaAs was not reported up until now. With the increase in applied potential, the transverse dimensions of triangular pores increase, leading to the overlapping of pores. As a result, the non-etched island having the same triangular shape remains as an individual nanowire.

One can see from Figure 1a that high-aspect-ratio GaAs nanowires tend to cluster in bundles. Several effects may be influencing this behavior, such as the electrostatic interaction between nanowires, non-uniform distribution of germination centers for the initiation of anodization on the initial surface of the substrate, and the stirring of the electrolyte during anodization. Note that, the higher the aspect ratio of nanowires, the higher the probability of bundles formation.

**Figure 3:**
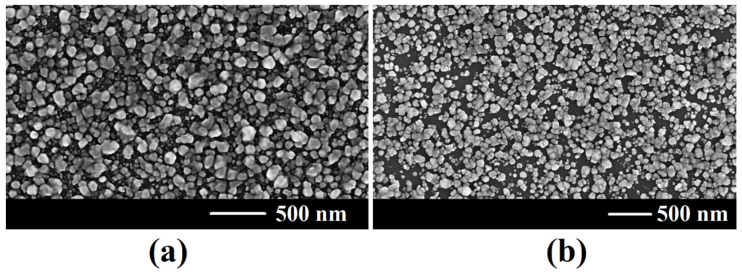
SEM images of electrochemically deposited Fe nanoparticles on bulk (001) GaAs (**a**) and (111)B (**b**) surface for 20 s.

The electrodeposition of Fe on the surface of both (001) and (111)B GaAs wafers for 20 s results in covering the sample surface with Fe nanoparticles that have sizes up to 100 nm, the coverage being total on (001) GaAs wafers and almost total on (111)B GaAs wafers (Figure 3).

**Figure 4:**
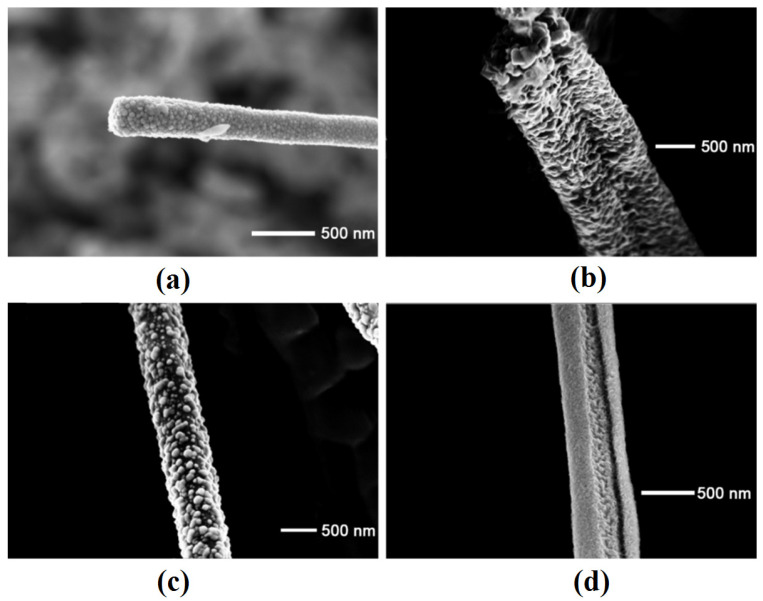
SEM images of electrochemically deposited Fe nanoparticles on GaAs nanowires produced on (001) GaAs after deposition for 20 s (**a**), and on GaAs nanowires produced on (111)B GaAs after deposition for 20 s (**b**), 15 s (**c**), and 10 s (**d**).

The coverage with Fe nanoparticles is also total on nanowires produced on both (001) GaAs (Figure 4a) and (111)B GaAs (Figure 4b) substrates after electrodeposition for 20 s, whereas the nanowires are only partially covered with Fe nanoparticles after deposition for 15 s (Figure 4c) and 10 s (Figure 4d). The deposition of nanoparticles on nanowires is quite uniform along the nanowire, the most uniform deposition being observed for nanowires produced on (001) substrates. The mean diameter of nanoparticles is within (30–120) nm depending on the deposition duration. The images of individual nanowires have been taken from those coming off the sample as a result of cleavage made for taking images in cross section.

The EDX analysis showed the stoichiometric composition of GaAs nanowires and the Fe composition of the metallic nanoparticles. The overall content of Fe in the GaAs core–Fe shell nanowire was found to increase from around 4% after deposition for 10 s to 7% after deposition for 15 s and to 10% after deposition for 20 s. Therefore, the produced arrays can be considered as being formed from GaAs core nanowires and Fe nanotube shells. The diameter of GaAs nanowires is around (250–500) nm, while the thickness of polycrystalline Fe nanotube walls is around 100 nm after deposition for 20 s, them being formed of separated Fe nanoparticles after deposition for 10 s and 15 s. Note that, in some cases, the nanowires were merged with one another as seen from Figure 4b,d, but the number of such cases is not significant.

**Figure 5:**
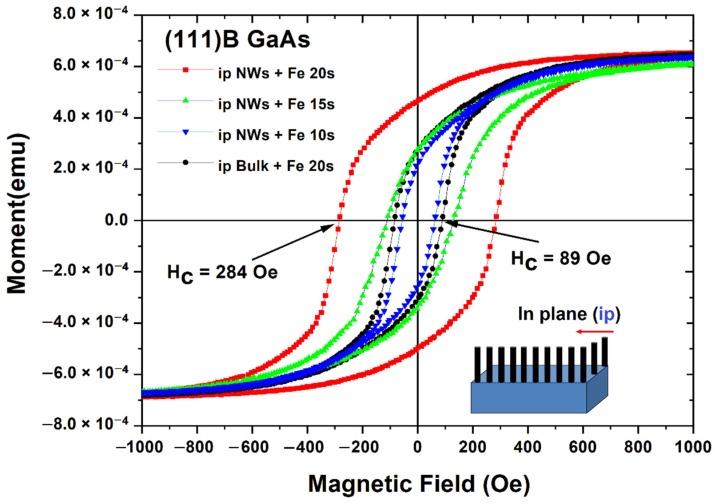
Hysteresis loops measured in in-plane configuration on nanowires array prepared on (111)B GaAs wafers compared with those measured on the substrate covered by a polycrystalline Fe film.

Figure 5 presents hysteresis loops measured in an in-plane configuration on a nanowires array prepared on (111)B GaAs. In such a case, the magnetic field is applied perpendicular to the nanowire array. The hysteresis loops measured on the substrate covered by a polycrystalline Fe film are shown for comparison. One can see that the coercivity of nanowires after Fe deposition for 10 s is nearly the same as that of the substrate after Fe deposition for 20 s, and it increases from 62 to 284 Oe when increasing the Fe deposition time from 10 to 20 s. The remanence ratio M_r_/M_s_ = RR (squareness) also increases from 0.35 to 0.70 with the increase in the deposition time.

**Figure 6:**
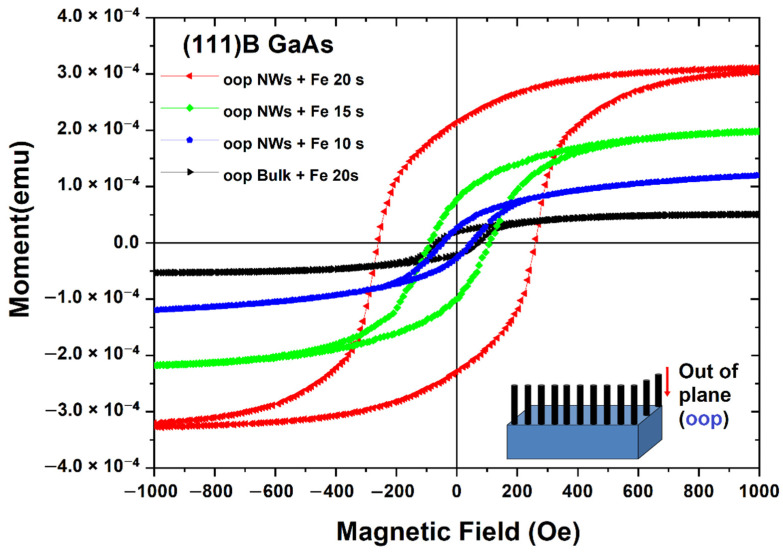
Hysteresis loops measured in out-of-plane configuration on nanowire arrays prepared on (111)B GaAs wafers compared with those measured on the substrate covered by a polycrystalline Fe film.

Similarly to the case of in-plane magnetization, for the out-of-plane magnetization, the coercivity of nanowires after Fe deposition for 10 s is nearly the same as that of the substrate after 20 s Fe deposition, and it increases from 46 to 260 Oe when increasing the Fe deposition time from 10 to 20 s (Figure 6). The remanence ratio also increases from 0.22 to 0.68 with an increase in the deposition time.

**Figure 7:**
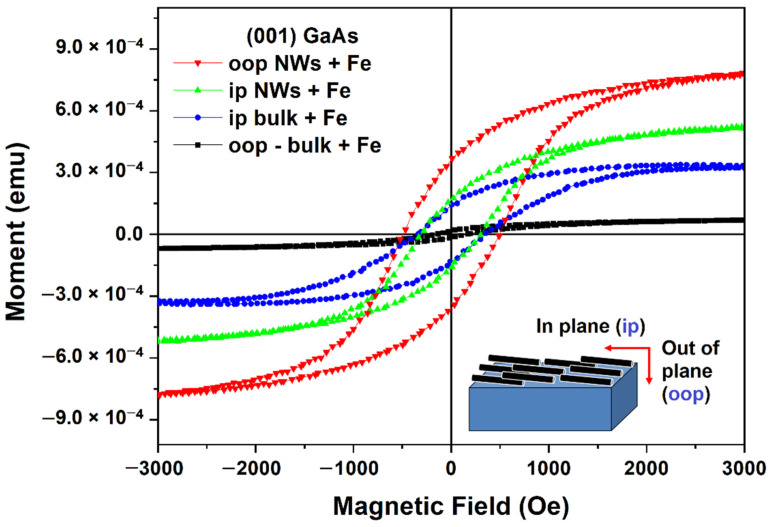
Hysteresis loops measured in in-plane and out-of-plane configurations on nanowires array prepared on (001) GaAs wafers compared with those measured on the substrate covered by a polycrystalline Fe film.

For the nanowires array prepared on (001) GaAs, i.e., for those oriented parallel to the wafer surface, all of the magnetic parameters (the saturation moment, the coercivity, and the remanence) are higher in the out-of-pale configuration, i.e., when the magnetic field is perpendicular to the nanowire array, compared to the case of in-plane magnetization (Figure 7). Note that the nanowires orientation in the case of the in-plane configuration is indefinite with respect to the direction of the magnetic field, i.e., it may be either parallel or perpendicular to the magnetic field, because the sample was not calibrated in this regard. If one compares the magnetic characteristics of the nanowires array with those of the magnetic film on the substrate, one can see that they do not differ significantly for the in-plane configuration, but the magnetic characteristics of the nanowires array are much higher than those of the film on the substrate for the out-of-plane configuration. In particular, the remanence ratio is two times higher, and the coercive force is three times higher.

The magnetic characteristics of the investigated samples are summarized in Table 1.

The comparison of the magnetic parameters of Fe nanoparticle films on GaAs substrates with different crystallographic orientations shows that the coercive force and the remanence ratio are higher for the in-plane compared to the out-of-plane configuration for both the crystallographic orientations of the substrate. On the other hand, the coercivity is higher by a factor of around two to four for the films deposited on (001) substrates compared to (111) substrates, which can be explained by a better coverage with Fe nanoparticles of the GaAs sample with (001) orientation, as seen from Figure 3.

**Figure 8:**
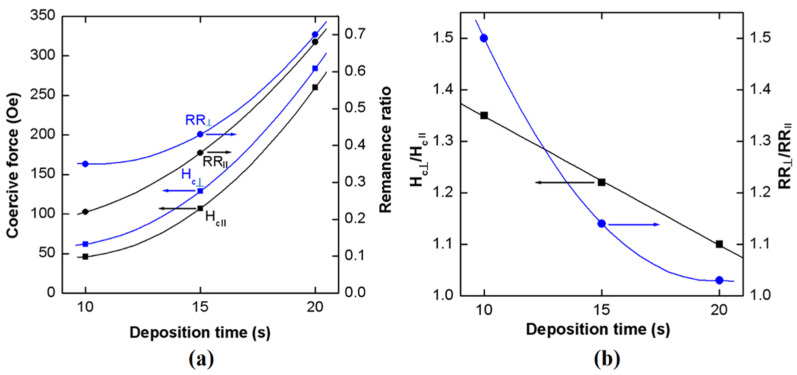
(**a**) Change in the coercive force (H_c_) and the remanence ratio (RR) with increase in the Fe deposition time on GaAs nanowires produced on (111)B substrates. (**b**) Dependence of the anisotropy of coercive force (H_c__⊥_/H_cII_) and remanence ratio (RR_⊥_/RR_II_) on the Fe deposition time. The lines are a guide for eyes.

The polycrystalline Fe nanotubes deposited on GaAs nanowires produced on (111)B substrates also demonstrate the increase in coercivity, as well as in the remanence ratio, when increasing their coverage with Fe nanoparticles as shown in Figure 8a. They also show some magnetic anisotropy with respect to the coercivity and the remanence ratio as shown in Figure 8b. On the other hand, whereas the magnetic parameters increase with an increase in the deposition time of Fe nanoparticles, the anisotropy of the coercive force (H_c__⊥_/H_cII_) decreases from 1.35 to 1.10 and the anisotropy of the remanence ratio (RR_⊥_/RR_II_) decreases from 1.50 to 1.03 with an increase in the deposition time of Fe nanoparticles from 10 s to 20 s. At the same time, the revealed anisotropy is different from that previously reported in small-diameter magnetic nanowires and nanotubes.

Both the coercivity and the remanence ratio of polycrystalline Fe nanotubes deposited on GaAs nanowires produced on (111)B substrates are higher when the magnetic field is oriented perpendicular to the nanowire axis (H_c__⊥_) than with the magnetic field oriented along the nanowire axis (H_cII_). The same is true for nanowires produced on (001) substrates, i.e., with nanowires oriented in-plane of the substrate. In such a case, the magnetic field is oriented perpendicular to the nanowire axis for the out-of-plane configuration, as illustrated in the inset of Figure 6. The ratio of H_c_(oop)/H_c_(ip) is 1.6, and the RR(oop)/RR(ip) is 1.43 for an Fe deposition time of 20 s.

It was previously shown that, in crystalline ferromagnetic Co nanowires with small diameters (26 nm), the coercive fields and the remanent ratio are higher for the magnetic field applied in the direction of the wire axis compared with the perpendicular direction [12]. It was also found that, in Ni nanotubes with a diameter of 35 nm, the coercivity is larger for the parallel orientation of the magnetic field than for the perpendicular orientation [26].

The difference between the magnetic anisotropy observed in the nanograin shells investigated in this study and those previously reported in small-diameter magnetic nanowires and nanotubes may indicate that the observed magnetic behavior of polycrystalline tubular structures is influenced both by the nanograin morphology and the nanotube geometry. As concerns previously reported data on larger nanowires and nanotubes, it was found that, even in more uniform morphology permalloy nanowires, the ratio of the longitudinal coercivity H_cII_ to transverse coercivity H_c__⊥_ can change from H_cII_/H_c__⊥_ > 1 to H_cII_/H_c__⊥_ < 1 when increasing either the length or the diameter of the nanowire. For instance, such a change was observed in nanowires with a length of 2 μm when the diameter was increased from 150 nm to 220 nm. A similar change occurred in nanowires with a diameter of 220 nm when the length increased from 1 to 2 μm, or in nanowires with a diameter of 250 nm when the length increased from 11 to 15 μm [27]. A similar behavior was observed in Ni and Co nanotubes, the ratio of H_cII_/H_c__⊥_ decreasing from 2.1 (Ni) and 1.4 (Co) nanotubes with a wall thickness of 12 nm, diameter of 35 nm, and length of 30 μm to the value of around 1 for nanotubes of both materials with the diameter of 160 nm [28]. The magnetic anisotropy in crystalline ferromagnetic nanowires and nanotubes, especially in those of a small diameter, has been previously discussed in terms of the magnetic reversal mechanisms. The magnetization crossover when increasing the geometrical dimensions was also suggested to be related to the magnetic reversal mechanisms. In ferroelectric nanotubes, there are basically two types of reversal mechanisms, namely vortex and transverse ones. The crossover between the two magnetization reversal modes is related to the movement of different types of domain boundaries: vortex wall and transverse wall [29,30]. Theoretical analysis predicted a crossover between two different magnetization reversal modes when increasing the wall thickness. It was found that, in thin tubes, the vortex domain wall is preferred, whereas, in thick tubes, the transverse magnetization reversal dominates. The situation seems to be more complicated in the case of polycrystalline tubular structures with nanograin morphology, since, in such a case, the movement of different types of domain walls is additionally influenced by the nanograin boundaries. This issue needs a more detailed investigation, which is the subject of a future publication.

## 4. Conclusions

The results of this study demonstrate possibilities to produce, using cost-effective electrochemical technologies, polycrystalline magnetic nanotube arrays oriented both in-plane and out-of-plane with the substrate, which enlarge opportunities to explore their magnetic properties and widen the area of their applications. It was found that the coercivity and remanence ratio of polycrystalline Fe nanotubes deposited on GaAs nanowires increase when increasing their coverage with Fe nanoparticles. The produced structures also exhibit some magnetic anisotropy with respect to the coercivity and the remanence ratio. The analysis of the obtained data in comparison with previous results on small-diameter magnetic nanowires and nanotubes suggests that the observed magnetic behavior of the produced structures is influenced both by the nanograin morphology and the nanotube geometry.

## Figures and Tables

**Table 1:** Saturation moment, remanence, and coercive forces for electrochemically deposited Fe on bulk and porous GaAs samples for in-plane and out-of-plane VSM measurements.

Crystallographic Orientation	Sample Description	Duration of Fe Deposition (s)	Saturation Moment, M_s_ (emu) × 10^−4^		Remanence, M_r_ (emu) × 10^−4^		Coercive Forces, H_c_ (Oe)	
			ip	oop	ip	oop	ip	oop
(111)B GaAs	Bulk/Fe	20	6.50	0.52	2.67	0.19	89	71
	Nanowires/Fe	10	6.42	1.2	2.25	0.26	62	46
		15	6.16	2.0	2.67	0.76	129	107
		20	6.60	3.1	4.65	2.1	284	260
(001) GaAs	Bulk/Fe	20	3.32	0.67	1.4	0.17	345	160
	Nanowires/Fe	20	5.1	7.75	1.68	3.62	308	500

## Data Availability

The data presented in this study are available on request from the corresponding authors.
